# Sleep duration and overactive bladder syndrome in US adults: a propensity score matching cohort study using NHANES 2005–2018

**DOI:** 10.3389/fneur.2025.1537796

**Published:** 2025-05-13

**Authors:** Jia-Jia Wang, Ai-Hong Jin, Ling-Min Hu, Xiu-Wei Tu, Xiao-Peng Huang, Li-Cai Mo

**Affiliations:** ^1^Department of Traditional Chinese Medicine, Taizhou Hospital of Zhejiang Province Affiliated with Wenzhou Medical University, Linhai, Zhejiang, China; ^2^Department of Nephrology, Ruian City Traditional Chinese Medicine Hospital, Ruian, Zhejiang, China; ^3^Department of Urology, Taizhou Cancer Hospital, Wenling, Zhejiang, China; ^4^Department of Urology, Taizhou Hospital of Zhejiang Province Affiliated with Wenzhou Medical University, Linhai, Zhejiang, China

**Keywords:** sleep duration, overactive bladder, cross-sectional study, NHANES, propensity score matching

## Abstract

**Objective:**

The association between sleep duration and overactive bladder (OAB) risk remains underexplored. The aim of this study was to assess this relationship in U.S. adults using National Health and Nutrition Examination Survey (NHANES) data.

**Methods:**

This cross-sectional study included 24,360 participants aged 20–80 years with OAB who completed the NHANES (2005–2018). NHANES Kidney Conditions-Urology Questionnaire data and the Overactive Bladder Symptom Score (OABSS) were collected. Sleep duration was self-reported by the participants. Propensity score matching (PSM) and multivariate logistic regression were employed to control for confounding variables, whereas a generalized additive model (GAM) was utilized to explore the nonlinear relationship between sleep duration and OAB.

**Results:**

Short sleep (<6 h) significantly increased OAB risk (OR 1.37; 95% CI, 1.21–1.55; *p* < 0.01). A U-shaped relationship between sleep duration and OAB was observed, suggesting that approximately 6 h of sleep is optimal for minimizing OAB risk. Additional risk factors for OAB included female sex, older age, higher BMI, lower education and income levels, hypertension, diabetes, and smoking.

**Conclusion:**

Both insufficient and excessive sleep durations were independently linked to increased OAB risk, and the optimal sleep duration to minimize OAB risk was approximately 6 h. These findings emphasize the importance of targeted prevention and intervention strategies focused on sleep and other modifiable risk factors for OAB. Further research is needed to better understand the biological mechanisms linking sleep duration and OAB.

## 1 Introduction

Overactive bladder syndrome (OAB) is characterized by lower urinary tract symptoms (LUTS), such as urgency, and often accompanied by urinary frequency and nocturia, with or without urgency urinary incontinence (1). As a functional bladder disorder, OAB imposes a substantial burden on patients, negatively affecting daily activities and psychological well-being. Epidemiological studies highlight the detrimental impact of OAB symptoms, which restrict routine activities and reduce overall health-related quality of life (2). Notably, OAB often leads to nocturnal disturbances, including urinary urgency and incontinence and nocturia, all of which are closely linked to sleep disruptions. For individuals with OAB, nocturnal bladder symptoms are commonly considered a primary factor in impaired sleep quality and disrupted sleep continuity (3).

Recent research has focused on the role of circadian rhythms in the development of LUTS (4). It is speculated that circadian core clock genes, such as Per2, Bmal1, Cry1/2, and Clock, may be involved in the pathophysiological changes observed in the development of OAB (5). In addition to its role in circadian regulation, sleep is widely recognized as a fundamental component of general health. Sleep hygiene — referring to behaviors that promote healthy sleep — plays a vital role in maintaining physical and mental well-being. Current guidelines recommend that adults aged 18–60 years should obtain at least 7 h of sleep per night to ensure adequate rest and recovery (6). Nonetheless, contemporary lifestyles, marked by shift work and recreational activities, often lead to insufficient sleep durations. In the United States, more than one-third of adults report routinely getting less than 7 h of sleep (7). Importantly, the negative effects of inadequate sleep extend beyond the urinary system, affecting multiple physiological processes, including immune regulation, nervous system stability, and metabolic homeostasis. For example, insufficient sleep has been linked to temporomandibular disorders (TMD), nervous system hyperstimulation, and disruptions in vitamin D metabolism (8). In this context, sleep deprivation has well-documented negative effects on health, particularly regarding nocturia within the spectrum of LUTS (2). This relationship may be bidirectional: frequent nocturia may cause sleep disturbances due to recurrent nocturnal awakening, and sleep disturbances may increase nocturia. Both conditions are linked to daytime fatigue, decreased quality of life, and increased risks of comorbidity (including depression, cardiovascular disease, and diabetes) and mortality (2, 9).

However, the relationship between sleep duration and the onset of OAB remains insufficiently understood, hindering the development of effective preventive measures. To explore this relationship, we analyzed data collected from the National Health and Nutrition Examination Survey (NHANES) collected from 2005 to 2018, aiming to better understand the interplay between sleep duration and OAB.

## 2 Materials and methods

### 2.1 Study population

In our study, we used data from seven two-year cycles of the National Health and Nutrition Examination Survey (NHANES), which was administered between 2005 and 2018. These years were selected on the basis of the availability of datasets containing relevant questions on sleep duration and urinary symptoms. The National Health and Nutrition Examination Survey (NHANES), managed by the Centers for Disease Control and Prevention (CDC), is a cross-sectional survey designed to collect nationally representative data through physical examinations, laboratory tests, and structured questionnaires.

Further details are available at https://www.cdc.gov/nchs/nhanes/about_nhanes.htm. Ethical approval for the use of NHANES data was granted by the Institutional Review Board (IRB) of the National Center for Health Statistics, and informed consent was obtained from all participants.

Since our study relies on existing NHANES data and does not generate any new data, additional ethical approval from external institutions is unnecessary. Participants aged 20–80 years who responded to the Kidney Conditions Questionnaire and reported sleep disturbances were included.

**Figure 1:**
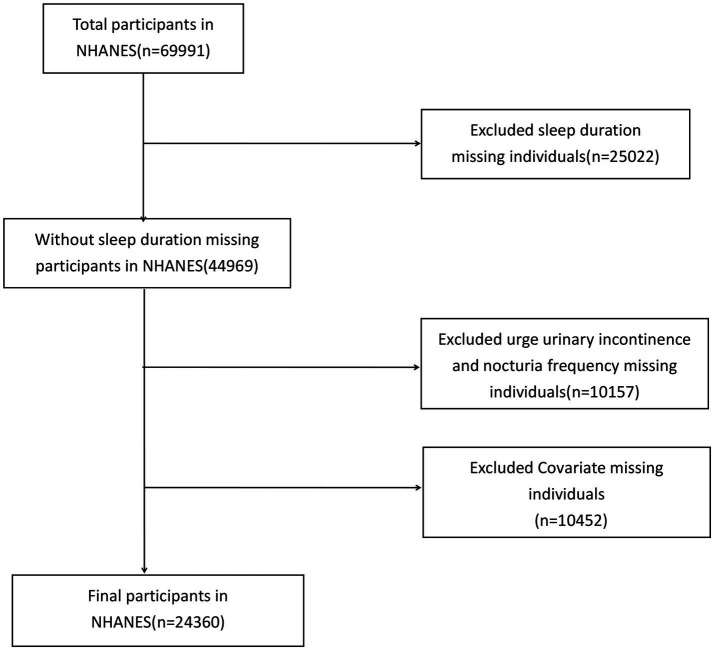
Flow chart of patient disposition.

Individuals without an OAB diagnosis or with missing sleep duration and covariate information were excluded. A total of 24,360 participants met the inclusion criteria (Figure 1).

### 2.2 Variable measurement: sleep duration and OAB assessment

Information on OAB symptoms, such as urgency urinary incontinence and nocturia, was gathered from the NHANES Kidney Conditions-Urology Questionnaire.

### 2.3 The severity of urgency urinary incontinence was assessed with two questions

“In the past 12 months, have you ever leaked or lost control of even a small amount of urine because you could not get to the toilet quickly enough owing to a sudden urge to urinate?”

“How often has this occurred?”

### 2.4 The severity of nocturia was evaluated via the following question

“In the past 30 days, on average, how many times per night do you wake up to urinate from the time you go to bed at night until the time you get up in the morning?”

To further quantify the symptoms of OAB, we employed the Overactive Bladder Symptom Score (OABSS) developed by Blaivas et al. (10). The detailed scoring criteria are provided in Table 1. The final OABSS for each NHANES participant was calculated by summing the scores for urgency urinary incontinence and nocturia. A total score ≥3 was considered indicative of OAB.

**Table 1:** Criteria for conversion of symptom frequencies recorded in NHANES and OABSS scores.

According to NHANES Score	According to OABSS Score
Urge urinary incontinence Frequency	Urge urinary incontinence score
Never	0
Less than once a month	1
A few times a month	1
A few times a week	2
Every day or night	3
Nocturia frequency	Nocturia score
0	0
1	1
2	2
3	3
4	3
5 or more	3

NHANES: National Health and Nutrition Examination Survey; OABSS: Overactive Bladder Symptom Score.

The participants underwent face–to-face interviews conducted by two well-trained researchers. They self-reported their typical sleep duration on workdays or weekdays and responded to the following question: “On a typical workday or weekday night, how many hours do you usually sleep?” Responses were recorded by the interviewers.

### 2.5 Other covariates

Demographic variables: Age, sex, race, annual household income, educational level, and marital status at the time of the interview were self-reported and obtained from NHANES demographic files.

Body mass index (BMI) data for participants were obtained from NHANES body measurements, which were conducted by trained health technicians at the Mobile Examination Center (MEC).

Tobacco Use: Smoking status was ascertained on the basis of two questions from the questionnaire section: (1) “Have you ever smoked at least 100 cigarettes in your entire life?” and (2) “Do you currently smoke?” Individuals who reported smoking every day or some days and had smoked more than 100 cigarettes were classified as “current smokers.” Those who reported that they were not currently smoking but had smoked more than 100 cigarettes in the past were classified as “former smokers,” whereas individuals who had smoked fewer than 100 cigarettes were classified as “nonsmokers” (4).

Other covariates, including hypertension, diabetes, and hypercholesterolemia, were selected on the basis of prior research and clinical experience.

### 2.6 Statistical analysis

Data are shown as medians (interquartile ranges) for continuous variables. Statistical differences between groups were assessed using chi-square tests for categorical variables and t tests for continuous variables. Logistic regression models were used to create propensity scores (PSs) on the basis of potential confounders. The confounding factors included sex (male/female), age, BMI (kg/m^2^), race (Mexican American/other Hispanic/non-Hispanic White/non-Hispanic Black/other race), education (less than high school/high school graduate/GED or equivalent/more than high school), marital status (married/widowed/divorced/separated/never married/living with partner), annual family income (under $20,000/$20,000 and above), and current smoking and drinking status (never/former smoker/current smoker; yes/no for current drinking).

A generalized additive model (GAM) was employed to explore the nonlinear relationship between sleep duration and OAB following propensity score matching (PSM) analysis. A two-piecewise linear regression model was applied to assess the potential threshold effect of sleep duration on OAB. The inflection point was determined automatically through a recursive method when a clear inflection point in the smoothed curve of OAB and sleep duration was observed.

All analyses were performed using R version 4.1.3, with two-tailed tests and a *p* < 0.05 indicating statistical significance. Logistic regression results were visualized using the R package “forestplot,” and related charts were created with the R packages “ggplot2” and “gtsummary.”

## 3 Results

### 3.1 Baseline characteristics of the study population

This study enrolled 24,360 participants for the baseline assessment (median age: 51 years [36, 65]; mean age: 49.8 ± 17.6 years; 51.5% female). More than 27.35% of the participants reporting OAB had a BMI over 30 kg/m^2^. Participants with OAB were more likely to have hypertension, diabetes, hypercholesterolemia, a history of smoking, lower educational levels, and lower annual family income. OAB was more prevalent among participants with shorter sleep durations. Table 2 provides a more detailed summary of participant characteristics.

**Table 2:** Baseline characteristics of the study population.

Variables	Overall	OABSS		*p* value
		No	Yes	
	*N* = 24,360	*N* = 19,129	*N* = 5,231	
Gender (%)				<0.001
Male	11,806 (48.5)	9,700 (50.7)	2,106 (40.3)	
Female	12,554 (51.5)	9,429 (49.3)	3,125 (59.7)	
Age (median [IQR])	51.00 [36.00, 65.00]	47.00 [34.00, 62.00]	63.00 [51.00, 73.00]	<0.001
BMI (median [IQR])	28.30 [24.50, 33.00]	27.90 [24.20, 32.40]	30.20 [25.85, 35.20]	<0.001
Race/ethnicity (%)				<0.001
Mexican American	3,275 (13.4)	2,577 (13.5)	698 (13.3)	
Other Hispanic	2,397 (9.8)	1864 (9.7)	533 (10.2)	
Non-Hispanic White	10,520 (43.2)	8,389 (43.9)	2,131 (40.7)	
Non-Hispanic Black	5,228 (21.5)	3,740 (19.6)	1,488 (28.4)	
Other Race	2,940 (12.1)	2,559 (13.4)	381 (7.3)	
Education (%)				<0.001
Less than high school	5,294 (21.7)	3,572 (18.7)	1722 (32.9)	
High school grad/GED or equivalent	5,478 (22.5)	4,222 (22.1)	1,256 (24.0)	
More than high school	13,588 (55.8)	11,335 (59.3)	2,253 (43.1)	
Marital status (%)				<0.001
Married	12,800 (52.5)	10,349 (54.1)	2,451 (46.9)	
Widowed	1968 (8.1)	1,154 (6.0)	814 (15.6)	
Divorced	2,773 (11.4)	1995 (10.4)	778 (14.9)	
Separated	782 (3.2)	544 (2.8)	238 (4.5)	
Never married	4,174 (17.1)	3,523 (18.4)	651 (12.4)	
Living with partner	1863 (7.6)	1,564 (8.2)	299 (5.7)	
Annual family income (%)				<0.001
Under $20,000	5,351 (22.0)	3,727 (19.5)	1,624 (31.0)	
$20,000 and Over	19,009 (78.0)	15,402 (80.5)	3,607 (69.0)	
Hypertension status (%)				<0.001
Yes	9,425 (38.7)	6,338 (33.1)	3,087 (59.0)	
No	14,935 (61.3)	12,791 (66.9)	2,144 (41.0)	
Diabetes status (%)				<0.001
Yes	3,595 (14.8)	2,125 (11.1)	1,470 (28.1)	
No	20,765 (85.2)	17,004 (88.9)	3,761 (71.9)	
High cholesterol (%)				<0.001
Yes	9,118 (37.4)	6,525 (34.1)	2,593 (49.6)	
No	15,242 (62.6)	12,604 (65.9)	2,638 (50.4)	
Smoke (%)				<0.001
Never	13,581 (55.8)	10,994 (57.5)	2,587 (49.5)	
Former smoker	6,154 (25.3)	4,544 (23.8)	1,610 (30.8)	
Current smoker	4,625 (19.0)	3,591 (18.8)	1,034 (19.8)	
Alcohol (%)				<0.001
Yes	18,202 (74.7)	14,555 (76.1)	3,647 (69.7)	
No	6,158 (25.3)	4,574 (23.9)	1,584 (30.3)	
Sleep duration (%)				<0.001
≥6 h	21,152 (86.8)	16,809 (87.9)	4,343 (83.0)	
<6 h	3,208 (13.2)	2,320 (12.1)	888 (17.0)	

NHANES, National Health and Nutrition Examination Survey; OABSS, Overactive Bladder Symptom Score; BMI, Body Mass Index.

### 3.2 Gender-stratified analysis of the association between sleep duration and OAB

The baseline characteristics revealed a significantly higher prevalence of OAB in females (59.7%) compared to males (40.3%) (*p* < 0.001). To further investigate this, we performed gender-stratified analyses to explore the relationship between sleep duration and OAB risk. As shown in Table 3, short sleep duration (<6 h) was significantly associated with a higher risk of OAB in both genders, with a stronger association in females. Specifically, females with short sleep duration had a 1.446-fold increased risk of OAB (OR = 1.446; 95% CI: 1.247–1.677; *p* < 0.001), while the corresponding OR in males was 1.254 (95% CI: 1.064–1.478; *p* = 0.007).

**Table 3:** Gender-stratified analysis of sleep duration and OAB risk.

Gender	Sleep Duration<6 h	95%CI lower	95%CI lower	*p* value
Male	1.254	1.064	1.478	0.007
Female	1.446	1.247	1.677	<0.001

### 3.3 Comparison between the low- and long-sleep duration groups before and after PSM

Given that factors such as sex, BMI, education, annual family income, hypertension status, diabetes status, high cholesterol, smoking, and alcohol consumption are closely associated with OAB and significantly differ between the low sleep duration (< 6 h) and long sleep duration (≥ 6 h) groups, we employed propensity score matching (PSM) to reduce these disparities. After PSM, we identified 3,208 matched pairs with comparable sex, BMI, education, annual family income, hypertension status, diabetes status, high cholesterol, smoking, and alcohol consumption (Table 4).

**Table 4:** Comparison between low sleep duration group and long sleep duration group before and after propensity score matching (PSM).

Variables	Before matching		*p* value	After matching		*p* value
	≥6 h	<6 h		≥6 h	<6 h	
	*N* = 21,152	*N* = 3,208		*N* = 3,208	*N* = 3,208	
Gender (%)			0.021			1
Male	10,190 (48.2)	1,616 (50.4)		1,616 (50.4)	1,616 (50.4)	
Female	10,962 (51.8)	1,592 (49.6)		1,592 (49.6)	1,592 (49.6)	
Age (median [IQR])	51.00 [36.00, 65.00]	51.00 [37.00, 63.00]	0.353	51.00 [35.00, 65.00]	51.00 [37.00, 63.00]	0.853
BMI (median [IQR])	28.20 [24.50, 32.80]	29.40 [25.10, 34.60]	<0.001	29.30 [25.20, 34.10]	29.40 [25.10, 34.60]	0.892
Race/ethnicity (%)			<0.001			<0.001
Mexican American	2,928 (13.8)	347 (10.8)		352 (11.0)	347 (10.8)	
Other Hispanic	2048 (9.7)	349 (10.9)		281 (8.8)	349 (10.9)	
Non-Hispanic White	9,416 (44.5)	1,104 (34.4)		1,348 (42.0)	1,104 (34.4)	
Non-Hispanic Black	4,128 (19.5)	1,100 (34.3)		785 (24.5)	1,100 (34.3)	
Other Race	2,632 (12.4)	308 (9.6)		442 (13.8)	308 (9.6)	
Education (%)			<0.001			0.834
Less than high school	4,480 (21.2)	814 (25.4)		828 (25.8)	814 (25.4)	
High school grad/GED or equivalent	4,663 (22.0)	815 (25.4)		825 (25.7)	815 (25.4)	
More than high school	12,009 (56.8)	1,579 (49.2)		1,555 (48.5)	1,579 (49.2)	
Marital status (%)			<0.001			<0.001
Married	11,361 (53.7)	1,439 (44.9)		1,545 (48.2)	1,439 (44.9)	
Widowed	1702 (8.0)	266 (8.3)		280 (8.7)	266 (8.3)	
Divorced	2,293 (10.8)	480 (15.0)		364 (11.3)	480 (15.0)	
Separated	612 (2.9)	170 (5.3)		100 (3.1)	170 (5.3)	
Never married	3,586 (17.0)	588 (18.3)		639 (19.9)	588 (18.3)	
Living with partner	1,598 (7.6)	265 (8.3)		280 (8.7)	265 (8.3)	
Annual family income (%)			<0.001			0.615
Under $20,000	4,461 (21.1)	890 (27.7)		871 (27.2)	890 (27.7)	
$20,000 and Over	16,691 (78.9)	2,318 (72.3)		2,337 (72.8)	2,318 (72.3)	
Hypertension status (%)			<0.001			0.707
Yes	7,981 (37.7)	1,444 (45.0)		1,460 (45.5)	1,444 (45.0)	
No	13,171 (62.3)	1764 (55.0)		1748 (54.5)	1764 (55.0)	
Diabetes status (%)			<0.001			0.218
Yes	3,035 (14.3)	560 (17.5)		599 (18.7)	560 (17.5)	
No	18,117 (85.7)	2,648 (82.5)		2,609 (81.3)	2,648 (82.5)	
High cholesterol (%)			0.051			0.818
Yes	7,867 (37.2)	1,251 (39.0)		1,241 (38.7)	1,251 (39.0)	
No	13,285 (62.8)	1957 (61.0)		1967 (61.3)	1957 (61.0)	
Smoke (%)			<0.001			0.279
Never	11,999 (56.7)	1,582 (49.3)		1,569 (48.9)	1,582 (49.3)	
Former smoker	5,389 (25.5)	765 (23.8)		816 (25.4)	765 (23.8)	
Current smoker	3,764 (17.8)	861 (26.8)		823 (25.7)	861 (26.8)	
Alcohol (%)			<0.001			0.78
Yes	15,888 (75.1)	2,314 (72.1)		2,325 (72.5)	2,314 (72.1)	
No	5,264 (24.9)	894 (27.9)		883 (27.5)	894 (27.9)	

PSM, Propensity Score Matching; BMI, Body Mass Index.

### 3.4 Multivariate analysis

**Figure 2:**
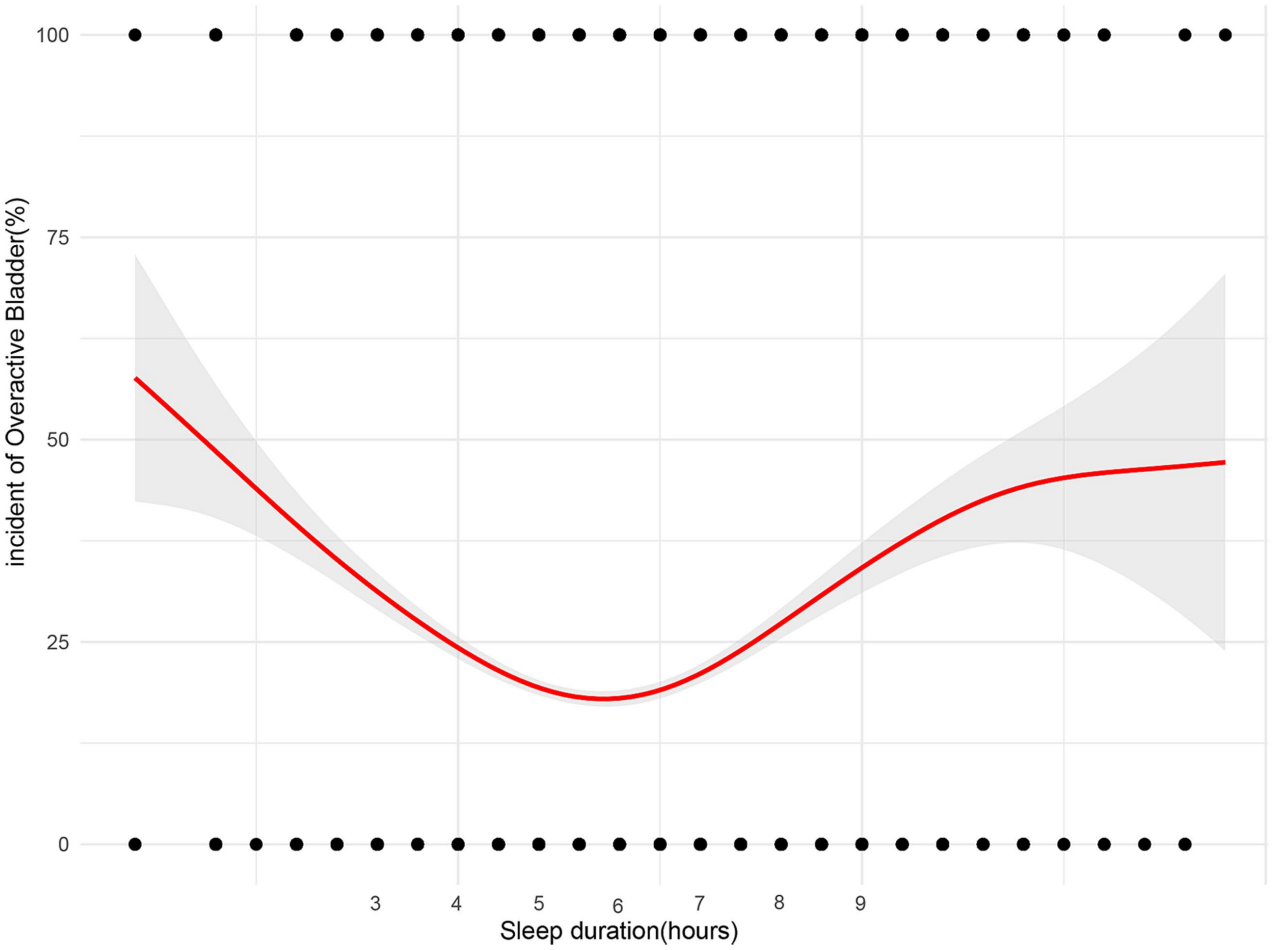
Forest plots depicting the associations between various demographic, socioeconomic, and health-related factors with sleep duration and the risk of Overactive Bladder (OAB). Each plot presents the odds ratios (OR) and corresponding 95% confidence intervals (CI) for individual variables. Statistically significant associations are marked by *p*-values < 0.05. The horizontal line at OR = 1 represents the reference point, indicating no effect, with factors whose confidence intervals do not cross this line being significantly associated with OAB risk.

After PSM, multivariable logistic regression identified sex, age, BMI, education level, marital status, annual family income, hypertension status, diabetes status, smoking, and sleep duration as independent risk factors for OAB. A sleep duration < 6 h was a significant risk factor for OAB. Patients with <6 h of sleep had a 1.325-fold greater risk of OAB than those with ≥ 6 h of sleep (OR 1.325; 95% CI, 1.169–1.501; *p* < 0.001; Figure 2). In the forest plot analysis, several significant associations were identified: females had a 1.727-fold greater risk of OAB than males did, and each additional year of age increased OAB risk by 4.2%. Higher education levels were associated with a decreased risk of OAB. Participants with an annual income > $20,000 had a 75.5% lower risk of OAB than those with an annual income < $20,000. Compared with hypertension, the absence of hypertension was associated with an 80.8% lower risk of OAB. Participants without diabetes had a 58.0% lower risk of OAB than those with diabetes did. Compared with nonsmokers, current smokers had a 46.5% increased risk of OAB (Figure 2).

**Figure 3:**
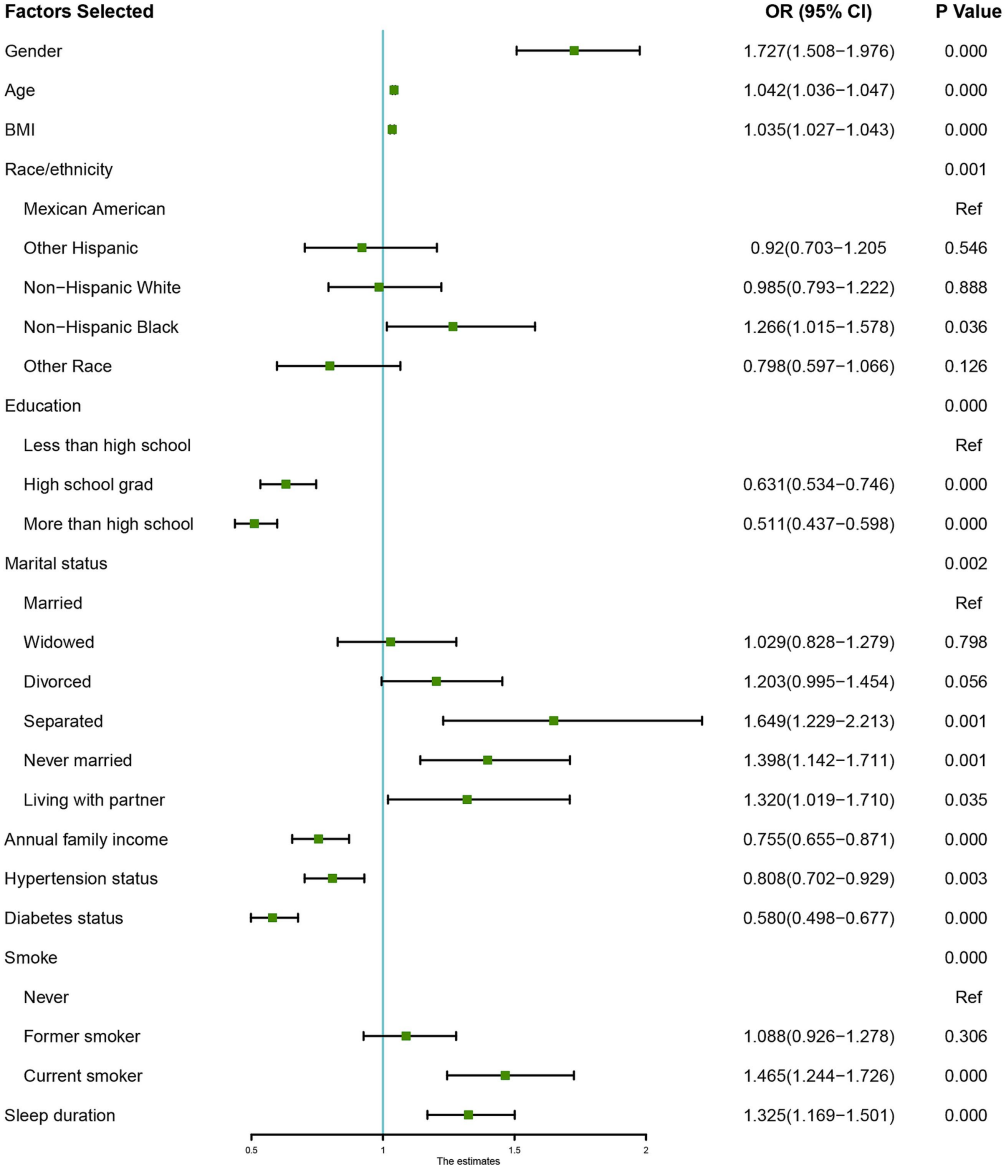
Non-linear relationship between sleep duration and the risk of Overactive Bladder (OAB). The plot illustrates the U-shaped association, with both short (<6 h) and long (>9 h) sleep durations showing increased OAB risk. The optimal sleep duration for minimizing OAB risk is approximately 6 h, as indicated by the curve’s lowest point.

In further analyses, a GAM was utilized to investigate the relationship between sleep duration and the incidence of OAB (Figure 3), revealing a nonlinear relationship between the two variables. Piecewise linear regression (Table 5) revealed that a sleep duration < 6 h was inversely related to the development of OAB [OR = 0.72 (0.68, 0.76), *p* < 0.001]. However, a sleep duration ≥ 6 h was associated with an increased incidence of OAB [OR = 1.32 (1.26, 1.38), *p* < 0.001]. On the basis of this analysis, a sleep duration of 6 h appears to be optimal for minimizing the risk of developing OAB.

**Table 5:** The result of two-piecewise linear regression model.

The turning point of sleep duration (h)	OR, 95%CI	*p*
Sleep duration < 6	0.72 (0.68, 0.76)	< 0.001
Sleep duration ≥6	1.32 (1.26, 1.38)	< 0.001
P for log likelihood ratio test		< 0.001

GAM, Generalized Additive Model; OR, Odds Ratio; CI, Confidence Interval.

## 4 Discussion

Our research demonstrated that sleep duration independently influences the development of OAB, revealing a U-shaped association after adjusting for confounding factors via PSM. The optimal sleep duration for minimizing OAB risk is approximately 6 h. Both short and long sleep durations were linked to an increased risk of OAB. Additional risk factors included sex, age, BMI, education, income, hypertension, diabetes, and smoking.

Our gender-stratified analysis found that short sleep duration is linked to a higher risk of OAB, with a stronger association in females. Several factors may explain this difference. Estrogen fluctuations during perimenopause and postmenopause can impair bladder function, increasing OAB risk in women (11). Additionally, females are more likely to experience sleep disturbances, which worsen nocturnal bladder symptoms (12). Anatomically, a shorter urethra and weaker pelvic support in females may also contribute to OAB (13). Furthermore, higher levels of stress, anxiety, and depression in women are known to exacerbate both sleep issues and OAB (14). These findings emphasize the need for gender-specific approaches in addressing sleep and OAB management. In addition, recent research has suggested that central sensitization-characterized by enhanced responsiveness of the central nervous system-may contribute to the presentation of symptoms in individuals with sleep disorders, including those with OAB (15). This mechanism may amplify the perception of bladder discomfort or urgency, particularly in those with chronic sleep disturbances.

OAB is a common urological condition that significantly disrupts daily activities due to its complex and multifactorial pathophysiology. A growing body of evidence suggests that factors such as age, diabetes, obesity, psychological and behavioral changes, and sleep disturbances contribute to OAB onset and progression (12, 16, 17). Sleep disturbances are associated with a greater risk of daytime urinary symptoms, which impact both the storage and micturition phases (18). Many OAB patients also experience sleep disturbances (12, 19). In women with OAB, nocturnal bladder symptoms are often considered the primary cause of poor sleep quality or sleep disruption (20). Women with insomnia may be more sensitive to bladder filling due to hyperarousal, resulting in more frequent nocturnal awakenings for urination. Conversely, they may awaken for other reasons and subsequently experience the urge to urinate, prompting bathroom visits (21). Furthermore, adequate sleep is essential for immune system homeostasis and for stabilizing the detrusor muscle of the bladder. Obstructive sleep apnea triggers cell survival signaling pathways, leading to oxidative stress in the bladder, as evidenced by increased malondialdehyde and advanced oxidation protein products. This oxidative stress contributes to detrusor muscle instability and exacerbates OAB symptoms (22). This study highlights the significant role of sleep duration in OAB risk. A U-shaped curve was observed, suggesting that both excessive and insufficient sleep duration increase OAB risk. This relationship remains robust even after controlling for potential confounding variables through PSM.

Despite the well-established association between sleep duration and OAB, the underlying biological mechanisms that explain why approximately 6 h of sleep may be optimal for reducing OAB risk remain unclear. One possible explanation involves the regulation of circadian rhythms, which are known to govern various physiological processes, including bladder function. Bladder muscle cells possess an intrinsic circadian clock regulating connexin 43 (Cx43), which enhances intercellular electrical and chemical transmission, decreases bladder capacity and increases urinary frequency, mimicking OAB symptoms (23). Sleep plays a key role in regulating circadian rhythms, with the transcription factor BMAL1/Mop3 being a pivotal regulator of the sleep–wake cycle in mammals (24). Disruptions in BMAL1/Mop3 can result in sleep fragmentation, increased electroencephalogram (EEG) delta wave activity, and reduced compensation for acute sleep loss (25). Deficiencies in the circadian rhythm gene BMAL1/Mop3 may alter the excitability of histaminergic neurons, affecting the brain’s biological clock and bladder function (26). This alteration could lead to abnormal bladder activity, potentially contributing to OAB development (27). Notably, sleep duration is a crucial component in maintaining circadian rhythm stability. Both insufficient and excessive sleep can disturb this homeostasis, thereby affecting bladder function. In addition, inflammatory and oxidative stress pathways may serve as important mediators linking abnormal sleep duration to bladder dysfunction. Both short and long sleep durations have been associated with immune dysregulation, elevated inflammatory responses, oxidative stress, hormonal imbalance, and psychological disturbances, all of which may contribute to the development and progression of OAB (28, 29). Sleep disturbances and acute sleep deprivation are linked to elevated levels of proinflammatory cytokines, such as prostaglandin E2 (PGE2), interleukin-6 (IL-6), tumor necrosis factor-alpha (TNF-*α*), and C-reactive protein (CRP), increasing susceptibility to infections and inflammation (30–32). The pathophysiology of OAB may involve dysregulated production of inflammatory mediators such as PGE2, leukotrienes, and CRP, indicating a potential inflammatory component in OAB development (33–35).

Moreover, individuals with sleep disorders frequently present with metabolic abnormalities (36). Previous studies have shown that males with metabolic syndrome are more likely to develop nocturia and impaired bladder emptying (37). Evidence indicates that obesity is an independent risk factor for OAB (38, 39). Endoplasmic reticulum stress in the bladder may lead to insulin resistance, potentially contributing to OAB development (40). Therefore, metabolic processes may mediate the link between sleep disturbances and OAB. The link between sleep duration and OAB is complex and requires further investigation.

In addition to sleep duration’s impact on OAB risk, the bidirectional nature of the relationship should be considered. Nocturia, characterized by the need to wake at night to urinate, is a prevalent symptom of OAB can lead to sleep deprivation and poor sleep quality (41). This sleep disruption can result in daytime fatigue, mood changes, and impaired cognitive function. Conversely, inadequate sleep may exacerbate OAB symptoms, creating a cyclical pattern where each condition perpetuates the other (12, 42). Therefore, both the quality and duration of sleep should be considered in future studies exploring OAB and its management. Among various aspects of sleep quality, sleep fragmentation — referring to repeated interruptions or arousals during sleep — has become an increasingly common issue in modern society. Fragmented sleep not only impairs sleep continuity and reduces restorative sleep stages but also adversely affects immune function, metabolic balance, and emotional regulation (43). These negative consequences may, in turn, increase the susceptibility to OAB and other chronic conditions.

Our multivariate analysis revealed that high income, higher education and abstaining from alcohol and smoking are protective factors against OAB. Consistent with previous studies, our research revealed a positive correlation between lower socioeconomic status (lower levels of education and income) and higher OAB incidence (44, 45). Individuals with higher levels of education are more likely to adopt health-promoting behaviors, whereas those with limited education are prone to poor dietary habits and toxin exposure (46). Smoking is a major public health challenge that can be prevented. Previous studies have consistently indicated that smoking is a risk factor for OAB (47, 48). Smoking is thought to affect OAB by disrupting nicotinic acetylcholine receptors in the nervous system (49). Furthermore, the antiestrogenic effects of nicotine on the bladder and urethra may lead to OAB and incontinence, suggesting a direct link between smoking and urological dysfunction (50). Alcohol consumption is associated with a higher risk of OAB (47), although some studies suggest a potential protective role of beer (51). In the context of alcohol consumption, both acute and chronic exposure are associated with dysregulation of serum hormone levels, including increased estrogen and decreased androgen levels (46). These hormonal changes may contribute to OAB development through various biological mechanisms, as elucidated by recent research (52). Experimental evidence suggests that testosterone may alleviate fibrotic changes in bladder tissue (53) and affect urethral mediator release, indicating that androgen deficiency could contribute to OAB (54).

This study is the first comprehensive cross-sectional investigation leveraging the NHANES database to examine the relationship between sleep duration and OAB. However, several limitations should be acknowledged. First, the cross-sectional design prevents the determination of causality regarding sleep duration and OAB. Second, the assessment of OAB relies on self-reported data and historical records, with the onset time of OAB being unknown. Third, sleep duration was based on a single self-reported question, limiting our ability to evaluate sleep patterns, quality, or specific sleep disorders. This approach may also introduce recall and social desirability bias, as participants could overreport what they perceive as ideal sleep duration. The absence of objective measures, such as actigraphy or wearable trackers, further limits accuracy. Additionally, sleep quality indicators such as fragmentation, efficiency, or deep sleep duration, were not included but may also affect OAB risk. Finally, environmental factors such as job stress, shift work, social pressure, noise, and climate—known to influence sleep and possibly worsen OAB—were not captured in the dataset and could not be controlled for. Future studies should incorporate objective sleep measures and account for these environmental and psychosocial variables to clarify the complex link between sleep and OAB.

## 5 Conclusion

Our cross-sectional study using the NHANES database revealed a U-shaped correlation between sleep duration and OAB, with an optimal duration of approximately 6 h. Moreover, our findings highlight the multifactorial nature of OAB, involving factors such as sex, age, BMI, education, income, hypertension, diabetes, and smoking. Future research should investigate the complex interactions between these factors and sleep duration to develop more precise prevention and treatment strategies for OAB.

These findings provide new evidence for integrating sleep health management into public health policies and chronic disease prevention strategies. Public health measures that emphasize appropriate sleep duration, combined with established lifestyle interventions, may help reduce the burden of OAB, particularly among high-risk groups. Future research should prioritize designing and assessing targeted sleep interventions to optimize bladder health and enhance population outcomes.

## Data Availability

The original contributions presented in the study are included in the article/Supplementary material, further inquiries can be directed to the corresponding authors.
